# The Impact of Marriage and Cohabitation on Financial Distress: Evidence from SHARE 2015 to 2022 for 16 European Countries

**DOI:** 10.21203/rs.3.rs-10795209/v1

**Published:** 2026-08-25

**Authors:** Hans Gevers

**Affiliations:** Estonian Business School

**Keywords:** financial distress, marriage, cohabitation, partnership, health, Europe, SHARE, longitudinal

## Abstract

In this article, the relevance of the known economic benefits of marriage and cohabitation is reconfirmed. Based on data from the Survey of Health, Ageing and Retirement in Europe (SHARE) of the period 2015 to 2022 and 16 European countries, the severity of financial distress is explained by relational, health, financial, as well as general characteristics. The study reveals that singles are less likely to report greater easiness to make financial ends meet. Furthermore, income and employment appear to enable overcoming financial distress, while liabilities and reduced health facilitate financial distress. From all countries, primarily Danes report greater easiness to make ends meet. Econometrically, the study relies on ordered and non-ordered, panel and non-panel logistic estimators, which integrate, if possible, calibrated longitudinal weights. Overall, the study illustrates that partnership relieves financial distress and that the relationship stability does matter.

## Introduction

A wide range of societal services, from public benefits to real estate development, ought to account for the characteristics of a society, e.g., the relationship status of citizens. The policies steering these services are mostly known by citizens and allow them to gain economic advantages. For being in a relationship, this may regard sharing of costs related to utilities, living areas, medication and food stocks, means of transportation, and the like. Nevertheless, obtaining these advantages implies long-term commitment to a partner, which may inherently be disadvantageous. The alternative is to remain single, which provides greater financial independence yet requires covering most of the burden of life alone. In this study, the relevance of the economic benefits of marriage or cohabitation is verified for singles and non-singles, married and non-married, in relation to financial distress. The aim of the study is to identify the determinants of financial distress as well as to scrutinise its relation to marital and relationship status. It contributes to the existing literature by revisiting the expectation of economic benefit resulting from marriage and cohabitation through the exploration of a large dataset across a little less than a decade. Furthermore, the study aims to support or refute this expectation and, as such, contributes to a certain extent to Grounded Theory (GT). This theory can be considered as anchored in the philosophical schools of positivism, hermeneutics, and pragmatism (Åge, 2011). The quantitative nature of this study is regarded as useful for “both verification and generation of theory”, as supported by Glaser and Strauss (2017). The study itself does strictly not fit in GT as it neglects its most typical characteristic, i.e. theoretical sampling. Nevertheless, the study identifies influences that hold over time and may be informative directly for policymaking and indirectly for theory building. The following sections discuss the existing literature, data, methodology, and results. The study is ended with a discussion and conclusion.

## Literature Review

As a significant inquirer, Gallup records in the U.S. a 20 percentage point well-being advantage for the married compared to the never married. Furthermore, a thriving effect occurs as well-being positively relates to higher marriage rates, which, in turn, relate to lower deaths of despair (Rothwell, 2024). This finding is anchored in the commonly known interrelatedness of health, wealth, and social participation. The subsequent paragraphs highlight several findings provided by the existing literature that support the relevance of these influences.

Marriage and cohabitation relate to better health behaviours than being single, as stated by Pollard and Harris (2013). They suggest that durable marriages are most beneficial for health, given their underlying commitment. Moreover, poorer mental health appears to stimulate engagement in cohabitation, and the extra income resulting from cohabitation is an additional protection for women. Stimpson, Wilson, and Peek (2012) similarly find that higher income triggers unmarried to report, just like married persons, fewer days of inactivity in comparison to unmarried poor persons. Stimpson et al.’s findings for the United States are challenged by Averett, Argys, and Sorkin (2013), who find that, besides positive health effects, Canadian married men also bear the explicit negative effects of being overweight triggered by marriage. Besides this physical effect, Hu and Coulter (2025) relate partnership to mental health, and find that among older people, males mentally benefit from moving from a living-apart-together relationship to marriage. However, they highlight that exiting the former relationship is much less mentally harmful than exiting cohabitation or marriage. This negative effect of exiting is expanded by Couch, Tamborini, and Reznik (2015). They follow divorced men for 20 years and notice that, nearing the end of the observation period, continuously divorced men are more likely to report work limitations and subsequently rely on disability benefits. They suggest that the loss of the economic benefits of marriage is an important driver of this finding. This loss may be countered by remarrying. Li, Jiang, Ge, and Cheng (2019) illustrate in their working paper that Chinese divorced who remarry appear to report a more favourable self-perceived health status. Overall, the continuation of the positive influence of marriage and cohabitation is expected to be conditioned by the stability of the union, as suggested by Kravdal, Wörn, and Reme (2023). Their Norwegian sample supports this finding for mental health. Moreover, they highlight that many of the positive mental changes precede the actual moment of cohabitation and marriage. On top of stability, homeownership may condition marriage and cohabitation. In Western societies, both are likely influenced by the American white picket fence dream. The latter suggests that the timing of, in particular, marriage depends on the availability of earnings to support a desired lifestyle. As such, homeownership or surplus income may act as an initial condition for marriage (Mamun, 2005). This condition may persist afterwards.

Given that it may be conditioned, the health benefit from marriage cannot be taken for granted. Unhappy marriages may turn the protective health effects of marriage into unhealthy influences, which overall result in a worse health status than would have been obtained when divorced or remained single (Lawrence et al., 2019). Additionally, the occurrence of the effects is disputable. Perelli-Harris et al. (2018) find the positive impact of marriage to vaporise when “childhood selection mechanisms, prior partnerships and childbearing” are accounted for. They find primarily a modest positive influence on health arising from living in any type of partnership instead of living single. Regardless of the disputation on the effect of marriage and cohabitation, it is serviced by governments. They may influence the marriage and cohabitation rate through tax incentives. E.g., a marriage bonus is available in Luxembourg, Germany, Poland, Belgium, Spain and Czechia, primarily according to household type and within-couple income differences. A marriage penalty may also occur, e.g., through pension reduction once retired, as occurs in Greece or Cyprus. Overall, cohabitation may be even more beneficial than marriage, e.g., as found in Cyprus or Italy (Christl et al., 2021). Another bonus arises from spousal cohabitation, i.e. people who married their only cohabiting partner. According to Vespa and Painter (2011), the latter note a “wealth accumulation that is twice as large as the rate of married individuals who never cohabited”. Yet, this finding only holds among whites and may be merely the result of marrying when reaching economic stability.

To conclude, the existing literature provides evidence for a health and economic benefit of marriage and cohabitation, yet also acknowledges its negative effects, such as being overweight or unhappily married. Moreover, marriage and likely cohabitation can be expected to be conditioned by long-term stability and preceding expectations regarding wealth. The literature review supports the expectation that financial distress as a mental health state is influenced to a given extent by partnership.

### Data

The analysis in this study relies on data from SHARE (Bergmann et al., 2019; Börsch-Supan et al., 2013). SHARE is a project of the European Research Infrastructure Consortium (ERIC), nowadays located in the SHARE Berlin Institute. SHARE supports policymaking through documenting a recurring health survey. This survey inquires European citizens about the effects of health, social, economic and environmental policies. For the analysis in this paper, data from SHARE Waves 6 to 9 (SHARE-ERIC, 2024a, 2024b, 2024c, 2024d) is referred to. The selected sample covers up to 58,731 unique respondents aged 50 to 89 years old in 2015, whom are carried forward to 2022. Table 1 on the following page summarises the variables in the sample.

In total, 17 variables are listed in Table 1. The first two variables cover the dependent variable which reflects to what extent the respondent can make ends meet. The categorical version rates Financial distress with 1 With great difficulty, 2 With some difficulty, 3 Fairly easily, and 4 Easily to make ends meet. The binary version bundles the first two categories as well as the last two categories to obtain a binary variable that rates Financial distress 0 Rather difficult, and 1 Rather easy to make ends meet. On top of the dependent variables, 16 independent variables are used in the regression analysis, of which the respondent’s unique identifier is not referred to in Table 1. The independents cover general characteristics, as well as relational, health, and financial features.

First, the general characteristic Age ranges from 50 to 96 years old with a mean of 70 years old. Years of education ranges from zero to 35 with a mean of 11 years of education. A last general characteristic reveals the Gender, being Male or Female. The sample appears well balanced based on Gender, given 44 per cent is Male, and 56 per cent is Female in 2015. Second, to reflect relational features, the variable Marital status covers four categories. The category 0 labels Officially with partner and combines the original SHARE-categories Married, living with spouse and Registered partnership. The category 1 Away from partner combines the original categories Married, not living with spouse and Divorced. The categories 2 Never married and 3 Lost partner are original. In order to capture the impact of change in marital status, as an indicator of stability, an additional variable reflects whether the status changed relative to the previous survey year. For the first survey year, no reference year is available and the marital status is assumed to be unchanged. The variable indicates if a married respondent is still married in a subsequent survey year, or if a non-married respondent got married in a subsequent survey year. It may in total regard multiple marriage entries and exits per respondent across the time horizon. The remaining situations are used as a baseline. Merely the relative impact of marriage exit versus entry is interpreted. The variable reports three categories, i.e. 0 No longer officially together, 1 All other situations, and 2 Newly officially together. Alternatively, to the Marital status, the Relationship status is used. Both status evidently strongly correlate as supported by their Spearman correlation of 0.854 (see Table 4 in Appendix). The Relationship status merely covers two categories, i.e. if the respondent is 0 Not single or 1 Single. Also, the change in the Relationship status, again indicating stability, is documented and constructed in the same way as the change in the Marital Status. The change in Relationship status is covered with 0 Hooked up, 1 Unchanged, and 2 Back single. Third, health features are included in the analysis as distress is expected to be dependent on a respondent’s health. Two variables can be integrated for this purpose, or the Self-perceived health status, or the Limitation in activities indicator. The former withholds five categories from 1 Excellent to 5 Poor, the latter reports 0 Not limited or 1 Limited. Fourth, financial features are included to narrow distress down to financial distress. The variables Liabilities and (Total household) Income cover the categories 0 None, 1 Below country average, and 2 Above country average. Both variables explicitly rely on the country averages, as these are expected to significantly differ between the selected countries. An additional binary variable Mortgage, being typically a loan for a home, is included as Gevers (2026c) illustrated that financial distress may be relieved by homeownership exit under certain circumstances. As such, the binary variable reports 0 No mortgage and 1 Mortgage. A last feature, Employment status, is in this study primarily regarded as financially relevant. It covers six categories, i.e. 0 Retired, 1 Employed or self-employed, 2 Unemployed, 3 Permanently sick, 4 Homemaker, and 5 Other (e.g., student or volunteer). Lastly, on top of all the micro-level variables, two macro-level variables may be integrated. The variable Country covers the 16 European countries, i.e. Austria, Germany, Sweden, Spain, Italy, France, Denmark, Switzerland, Belgium, Czech Republic, Poland, Luxembourg, Portugal, Slovenia, Estonia, and Croatia. Table 1 refers to the original SHARE-number allocated to each country. The variable Survey year reflects the survey years 2015, 2017, 2020, and 2022. The latter is preferred to be used as panel level variable in combination with the respondent’s unique identifier. To conclude, some variables required preprocessing. The variables Financial distress and Job situation originally hold a category Not applicable, which has been dropped. Total household income and Liabilities are winsorized at the 99th percentile to counter bias resulting from extreme values. Both preprocessing and recategorising result in a tailored dataset suitable for the aim of this study.

To provide additional insight into the composition of the data sample, three graphs are plotted.

**Figure 1:**
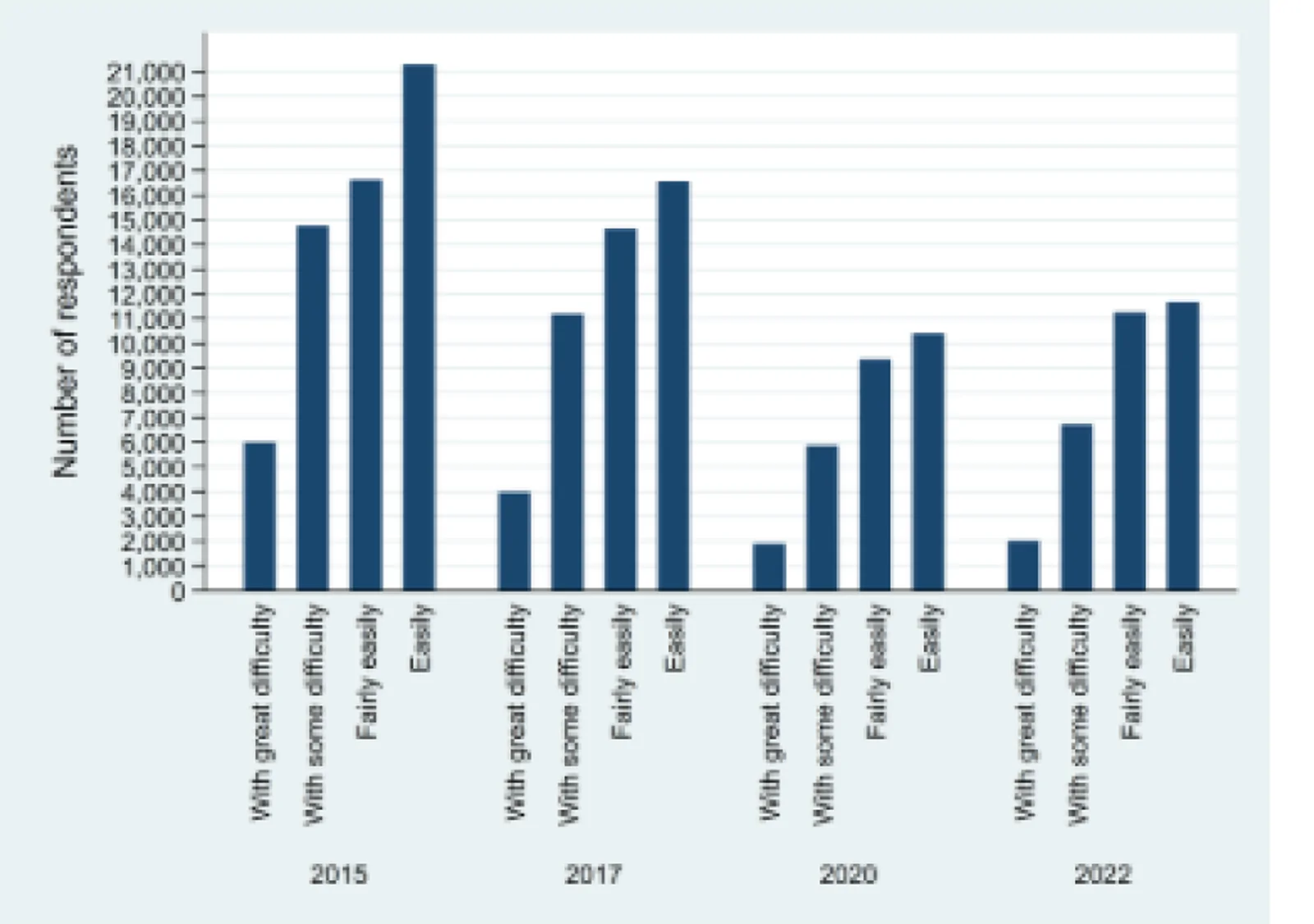
Number of Respondents per Financial distress level (make ends meet) and per Survey year

**Figure 2:**
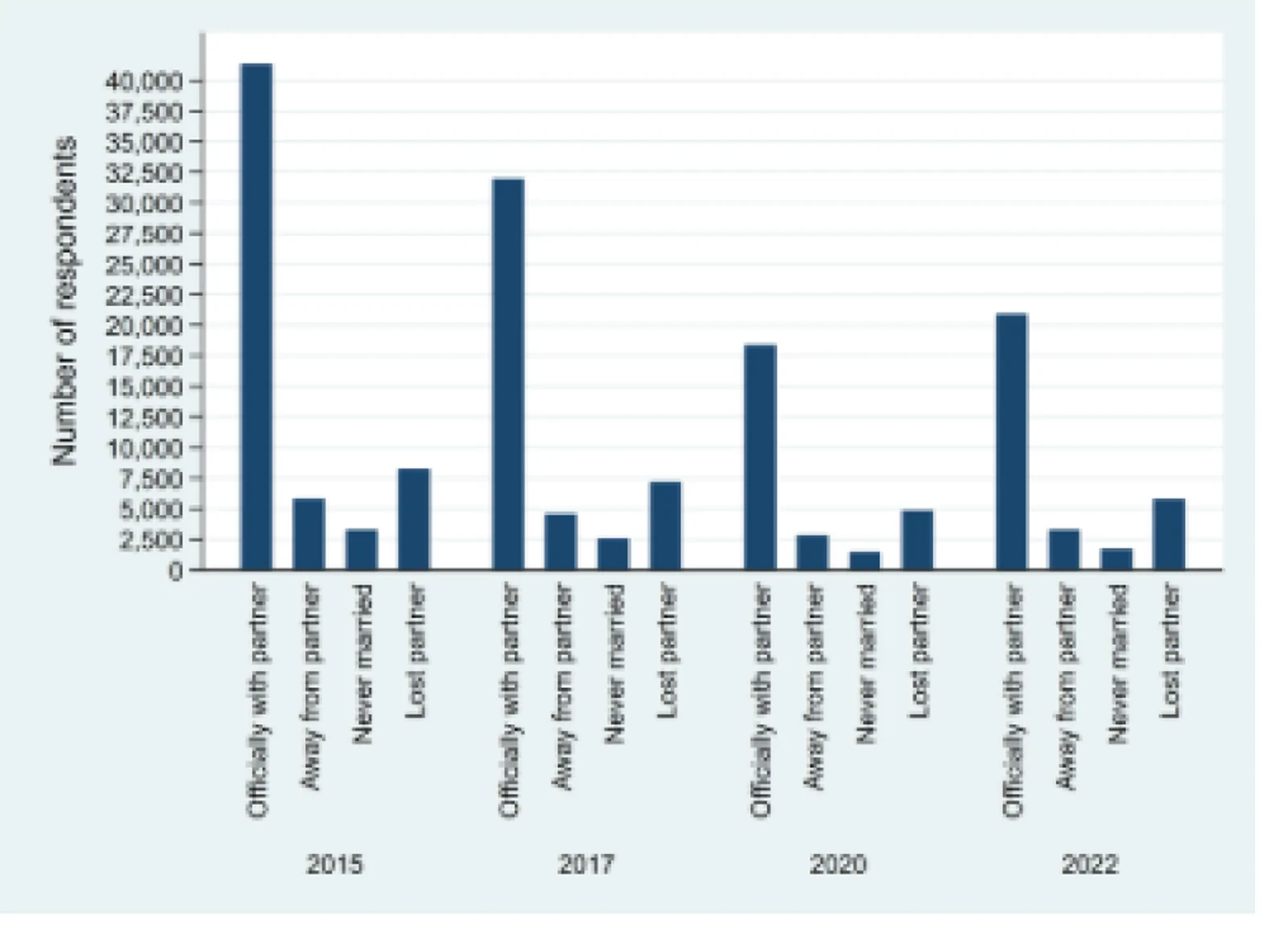
Number of Respondents per Marital status and per Survey year

**Figure 3:**
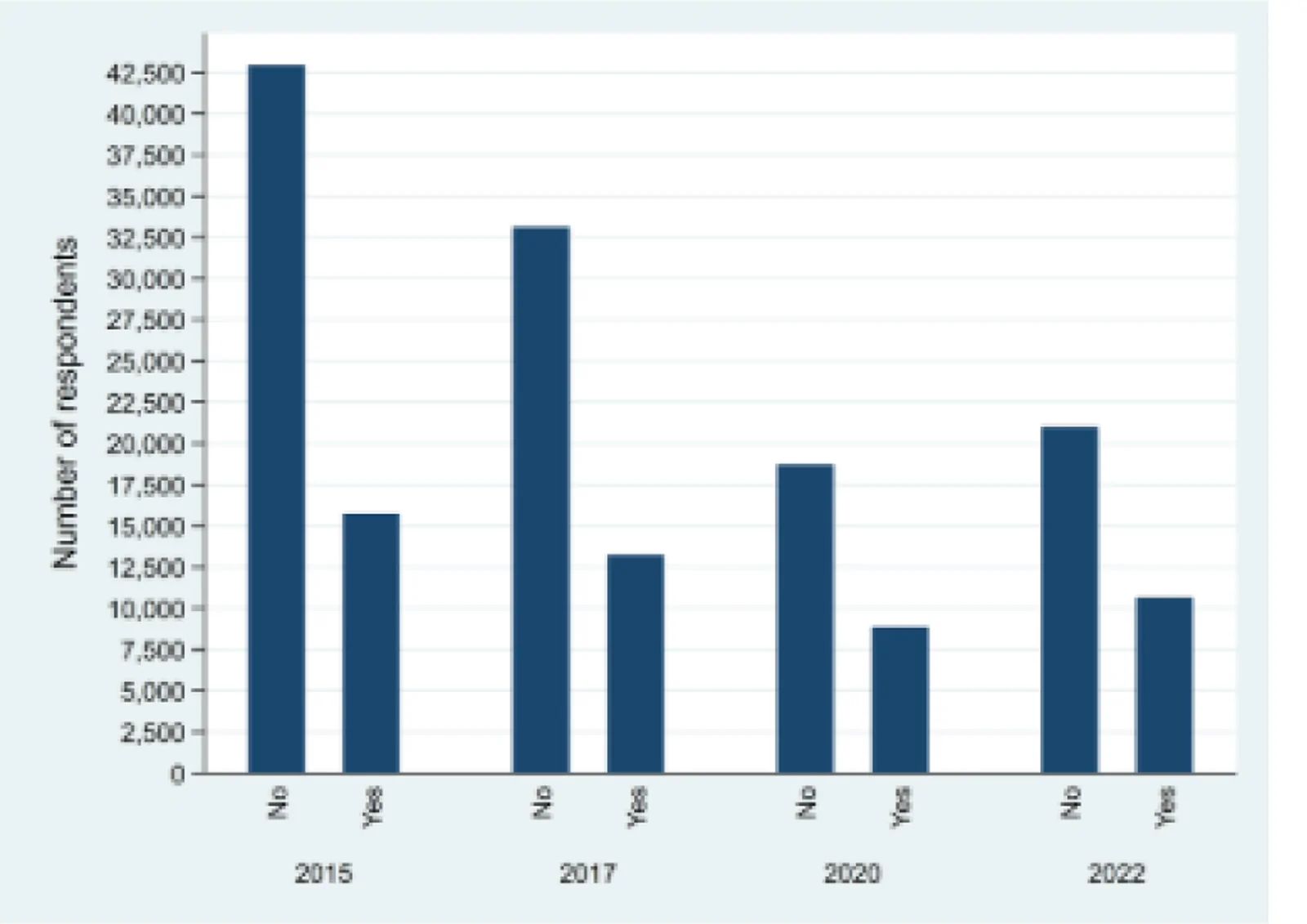
Number of Respondents per Relationship status and per Survey year

Figure 1 reveals the number of respondents per survey year in a given category of the dependent variable Financial distress. It shows that the sample is rather balanced, and that the majority of the respondents report, across years, being able to make ends meet fairly easily or easily. The figure, like the subsequent Figs. 2 and 3, illustrates that respondents are carried forward over time and that the sample shrinks over the years. Note that the lower number of observations in 2020 relative to 2022 is primarily due to the non-availability of observations for Portugal for 2020. This also applies to the subsequent Figs. 2 and 3, and is expected not to be an issue for the regression analysis.

Figure 2 documents the number of respondents per category of Marital status per survey year. Most of the respondents appear to be officially with a partner. Those who are never married are consistently the minority.

Lastly, Fig. 3 plots the number of respondents per Relationship status per survey year, and clearly polarises the concept of living together in comparison to the Marital status variable. Among the 50- to 89-year-olds in 2015, the minority is and remains single. To conclude, all variables are expected to be relevant for explaining financial distress, in particular, to enable isolating the influence of marital or relationship status.

## Methodology

The data analysis is completed in Stata 19.5 Basic Edition. It is a quantitative analysis which implements traditional econometric estimators. All estimators used are logistic estimators, given that the dependent variable Financial distress is consistently specified in categories. Four types of logistic estimators are implemented, covering Financial distress as a binary or categorical variable. A priori, panel estimators are preferred as they account for the fact that respondents are carried forward over survey years. The estimators rely on random-effects and random-effects ordered logistic models. Of the former, also the fixed-effects version is provided to enable taking the Hausman test. Despite that for logistic estimators this approach is not theoretically backed, thus may be inappropriate, it highlights the importance of verifying the preference for the fixed- or random-effects estimator. In this study, random effects are a priori more desirable to include as they can document the differences between time-invariant variables like the Country and Gender. To report a robust interpretation of the results, logistic and ordered logistic non-panel estimators are included, which both account for intragroup correlation to a certain extent, given that they report cluster standard errors. Yet, both estimators are not preferred, given that their odds do not account for the fact that the same respondents are carried forward over time. For three estimators, the integration of calibrated longitudinal weights is possible. The weights are constructed according to Pacifico’s (2014) procedure. The procedure uses Eurostat data of population and mortality, per year, country, age and gender. It aims to overcome issues with nonresponse, refreshment as well as attrition (De Luca & Rossetti, 2018). The calibrated longitudinal weights are, by theory, suitable for the ordered logistic panel estimator, yet are also implemented in the non-panel logistic and non-panel ordered logistic estimators. These weights are implemented and not the regular sampling weights to ensure that the respondents are reweighted in a similar way as done in the ordered logistic panel estimator. To conclude, the output of the estimators represents the average effect that holds ceteris paribus.

## Results

The output of the logistic estimators is reported in Tables 2 and 3. The former explains Financial distress with Marital status, the latter with Relationship status. Both integrate the same general characteristics as well as health and financial features. Table 2 on the following page documents the output of the logistic estimators for Financial distress in relation to Marital status.

The results in Table 2 reveal that of the general characteristics, only Age is consistently significant across estimators. As such, an increase in Age is expected to increase the odds of reporting less financial distress. This aligns with the expectation that at an older age, the financial capacity is more likely to be perceived as sufficient for the expected remaining life years. Similar to Age, Years of education is expected to positively influence the financial distress level despite that its influence is not confirmed by the fixed-effects estimator. Gender appears not to significantly impact the financial distress level.

Regarding the financial feature Income, one category appears informative across estimators, i.e. the category Above country average income. Soundly, those with a relatively higher income relate to higher odds of reporting less financial distress, or greater easiness to make ends meet. Liabilities appear most relevant, given that both categories consistently mark significance levels of p < 0.01. As reported by the fixed-effects and several non-panel estimators, those with below country average liabilities note slightly higher odds of reporting less financial distress than those with above country average liabilities. However, the inverse claim is supported by the random-effects estimators. As such, the difference between both is regarded as not that explicit, and subsequently primarily those without liabilities are expected to more likely report less financial distress. The last financial feature, Mortgage, captures the explicit effect of a debt that allows the debt issuer to seize the borrower’s assets. It reports lower significant odds, yet higher than those for liabilities, of reporting greater easiness with making ends meet. Lastly, the financial impact of the Employment status appears relevant across categories. Employed or self-employed come with higher odds of reporting greater easiness to make ends meet relative to the Retired. Unemployment consistently suggests to more likely end up in financial distress, an effect that is a little less explicit among the Permanently sick. The odds further increase for Homemakers and the category Other (with students, volunteers, and the like), yet remain lower than those of the Retired. For the categories Permanently sick and Other, the effects are not confirmed by the fixed-effects panel estimator.

For the concept of interest, covering marriage and cohabitation, the variable Marital status reveals a highly relevant lower odds of reporting less financial distress when the respondent went away from their partner. Similarly, yet less explicitly, the same reasoning leads to concluding that those respondents who have lost their partner or who are never married are less likely to report greater easiness to make ends meet relative to those officially with a partner. Interestingly, the year-on-year change in the Marital status, with all other situations as baseline, suggests that splitting up results in higher odds to make financial ends meet, and the fact of hooking up in lower odds. It is plausible that the economic benefit of official partnership is not instant and takes some time before thriving. Regardless of this suggestion, the results primarily support that stability in marriage is regarded as relevant for financial distress given that the main effect of being officially with a partner is beneficial for financial distress, while being newly being officially together with a partner appears less beneficial.

The last features relate to health and provide a clear indication that the less able also tend to end up in situations of financial distress. As such, those who are limited in activities report significant lower odds of making ends meet than their non-limited counterparts. Similarly, the worsening of the health status aligns with increasingly lower odds of reporting easiness to make financial ends meet. These findings can be explained by various phenomena, yet most evident is that stress is strengthened by physical inactivity and reduced mental resilience.

Lastly, the reliance on random-effects estimators is supported by their ability to document the effect of time-invariant variables like Country and Gender. Primarily, the differences between countries are of interest. The results tend to confirm the welfare dominance of Denmark followed by Sweden, Austria, Luxembourg and Switzerland as they report relatively higher odds of reporting greater easiness to make ends meet. Countries like Germany, France, Belgium, and Czechia appear to be in a mid-segment. Countries with a priori low odds of reporting less financial distress are Spain, Italy, Poland, Portugal, Slovenia, Estonia, and Croatia. Despite the fact that the findings roughly align with the commonly documented European divide (e.g., Gevers, 2026a), the difference may arise from a different perception of what financial distress implies. Besides the countries, the influence of the survey years is reported for the non-panel estimators. These dominantly report higher odds of reporting greater easiness with making ends meet relative to the base year, 2015. Mentionable is the year 2020, where the overall higher odds are a little lower relative to the previous year, 2017, as well as to the subsequent year, 2022. This slight decrease plausibly results from the COVID-19 pandemic.

To support the estimators used in Table 2, additional tests are included. As such, the Mundlak and Hausman test as well as the parallel regression assumption are additionally documented as illustrated by Gevers (2026b). Note that the tests are not theoretically supported for logistic panel estimators, implying that they may be inaccurate, even inappropriate. Nevertheless, the equations of panel logistic estimators (2) and (3) are used without Mundlak means to compare fixed- and random-effects estimators through a Hausman test. The tests reveal that the difference in coefficients is systematic for the estimators integrating the limitations with activities as well as for those integrating the self-perceived health status (respectively, (2) Chi squared 4406.16, p < 0.000 and (3) Chi squared 4287.66, p < 0.000). Additionally, the significance of the unit-specific time averages integrated in estimators (2) to (5) is tested. These averages appear jointly significant (respectively, (2) Chi squared 3412.72, p < 0.000; (3) Chi squared 3320.00, p < 0.000; (4) Chi squared 1344.74, p < 0.000; Chi squared 1433.42, p < 0.000). Both the Hausman and Mundlak tests provide a theoretically unsupported indication, which backs the preference for the fixed-effects estimators. Acknowledging this expectation, the results of the random-effects estimators are benchmarked against the other types of estimators to strengthen the robustness of the conclusions. Furthermore, given that the estimators (4), (5), (8), and (9) are ordered logistic estimators, the parallel regression assumption is tested based on estimator (8) without calibrated longitudinal weights. The assumption appears violated implying that the estimates of the ordered logistic estimators may be inaccurate. Testing the assumption for the panel estimators is currently not supported in the existing literature, implying that the concluded violation is not informative for estimators (2) to (5). Nevertheless, the non-ordered estimators are used for benchmarking and to counter any possible inaccuracy. Further details on all additional completed tests are available through the online materials (see Materials and Code availability).

On top of the influence of marriage, the impact of cohabitation is documented through the Relationship status, which reflects whether a respondent is single or not. Financial distress is related to this status in Table 3 on the following page.

The interpretation of the results of Table 2 also holds for the results documented in Table 3. This is quite sound given that merely two variables differ, namely the Marital status, which has been replaced by the Relationship status, as well as the year-on-year change in Marital status, which has been replaced by the year-on-year change in Relationship status. As such, the odds for the Relationship status and its change are of interest. Aligning with the economic benefits of marriage documented in Table 2, the Relationship status in Table 3 explicitly confirms, for singles, expected lower odds of reporting greater easiness with making ends meet. Similarly, the change in the Relationship status across the time horizon appears, like for the Marital status, to suggest that those who hooked up mark lower odds of reporting greater easiness to make financial ends meet. The positive influence on financial distress of becoming single is, in contrast to the findings in Table 2, merely backed by the random-effects estimators. Primarily, relationship stability appears relevant for financial distress as confirmed by the Change in Relationship as well as Marital status. Lastly, a given set of additional tests is completed with the same theoretical constraints as documented for Table 2. The Hausman tests, as well as the significant Mundlak means integrated in the estimators (2) to (5), suggest using fixed-effects estimators (Hausman, respectively, (2) Chi squared 4257.64, p < 0.000 and (3) Chi squared 4149.54, p < 0.000)(Mundlak, respectively, (2) Chi squared 3336.27, p < 0.000; (3) Chi squared 3242.47, p < 0.000; (4) Chi squared 1339.02, p < 0.000; Chi squared 1433.11, p < 0.000). Again, the results of the random-effects estimators are benchmarked against the other types of estimators to support accuracy. Similarly, the violated parallel regression assumption is overcome by relying on the non-ordered estimators as a benchmark for the interpretation of the results. Further details on all additional completed tests are available through the online materials (see Materials and Code availability).

## Discussion

This study confirms the importance of financial distress determinants like health, income, liabilities, and employment as already documented by Gevers (2026c). On top, it identifies the economic benefit of marriage and cohabitation by providing evidence that partnership positively influences financial distress. Nevertheless, the study is also limited. As both Hausman and Mundlak tests confirm a preference for fixed-effects estimators, applying the Mundlak correction as illustrated by Yair Mundlak (1978) on an logistic estimator may be desirable, yet using neither the tests nor the correction for logistic panel estimators is currently theoretically supported. Wooldridge (2010, pp. 610–625) provides some documentation for the application of the Mundlak-Chamberlain device to parametric models, like probit and logit estimators. In this study, this mathematical starting point is not further explored, yet its exploration is highly desirable to provide theoretical support. Additionally, the study is limited, given that many other intrinsic and extrinsic drivers may determine the occurrence of financial distress. For example, financial distress in relation to partnership may vary according to earlier experiences in life, the presence of children, and the like (Perelli-Harris et al., 2018). Overall, the study offers several opportunities for future research, from reproducing the findings with similar datasets to supporting the implementation of the Mundlak correction in logistic estimators with mathematical approaches.

## Conclusion

This article reveals that older people with a partner are more likely to report making ends meet. As such, it explicitly supports the economic benefit of marriage, cohabitation, or partnership. Furthermore, it identifies the positive influence of ageing and income, as well as the negative influence of liabilities, mortgages, and unemployment on financial distress. Additionally, financial distress is more likely when being limited or in poorer health. Geographically, more difficulties in making ends meet are expected in the Southern and Eastern European regions, which roughly confirms the existence of the European divide. Lastly, the study highlights the importance of stability in relationships or marriages for coping financial distress. Overall, partnership can be regarded as a remedy for financial distress.

## Supplementary Material

Supplementary Files

This is a list of supplementary files associated with this preprint. Click to download.

• Appendix.docx

## Figures and Tables

**Table 1:** Descriptives

Variable	Unique	Mean	Minimum	Maximum
Financial distress (binary)	2	-	0	1
Financial distress (categorical)	4	-	1	4
Age	47	69.584	50	96
Years of education	31	11.110	0	35
Gender	2		1	2
Marital status	4	-	0	3
Change in marital status	3	-	0	2
Relationship status	2	-	0	1
Change in relationship status	3	-	0	2
Self-perceived health status	5	-	1	5
Limited in activities	2	-	0	1
Liabilities	3	-	0	2
Mortgage	2	-	0	1
Total household income	3	-	0	2
Job situation	6	-	1	6
Country	16	-	11	47
Survey year	4	-	2015	2022

Number of observations: 164,331

**Table 2:** Estimation results for Financial distress in relation to the Marital status

VARIABLES	(1)	(2)	(3)	(4)	(5)	(6)	(7)	(8)	(9)
Age	1.112***	1.049***	1.051***	1.045***	1.049***	1.029***	1.032***	1.026***	1.030***
	(0.004)	(0.002)	(0.002)	(0.003)	(0.003)	(0.002)	(0.002)	(0.002)	(0.002)
Years of education	0.990	1.125***	1.116***	1.130***	1.122***	1.076***	1.071***	1.078***	1.073***
	(0.013)	(0.004)	(0.004)	(0.006)	(0.006)	(0.005)	(0.005)	(0.004)	(0.004)
Male (base)									
Female	-	1.010	0.992	1.030	1.022	1.020	1.012	1.021	1.013
		(0.026)	(0.025)	(0.042)	(0.041)	(0.036)	(0.036)	(0.028)	(0.027)
No income (base)									
Below country average income	1.003	0.860***	0.852***	0.895***	0.882***	1.022	1.024	1.023	1.024
	(0.023)	(0.018)	(0.018)	(0.030)	(0.029)	(0.045)	(0.045)	(0.037)	(0.037)
Above country average income	1.376***	2.589***	2.529***	2.575***	2.514***	3.430***	3.399***	3.247***	3.204***
	(0.040)	(0.067)	(0.065)	(0.103)	(0.101)	(0.173)	(0.173)	(0.127)	(0.126)
No liabilities (base)									
Below country average liabilities	0.648***	0.382***	0.386***	0.385***	0.389***	0.369***	0.376***	0.377***	0.384***
	(0.029)	(0.014)	(0.014)	(0.022)	(0.022)	(0.019)	(0.019)	(0.016)	(0.017)
Above country average liabilities	0.612***	0.416***	0.411***	0.392***	0.397***	0.340***	0.345***	0.400***	0.407***
	(0.037)	(0.021)	(0.021)	(0.029)	(0.029)	(0.027)	(0.027)	(0.024)	(0.025)
No mortgage (base)									
Mortgage	0.847***	0.858***	0.844***	0.728***	0.728***	0.764***	0.757***	0.769***	0.762***
	(0.043)	(0.033)	(0.033)	(0.041)	(0.041)	(0.047)	(0.048)	(0.034)	(0.034)
Officially with partner (base)									
Away from partner	0.606***	0.343***	0.349***	0.402***	0.414***	0.555***	0.565***	0.601***	0.608***
	(0.097)	(0.014)	(0.014)	(0.029)	(0.029)	(0.032)	(0.032)	(0.028)	(0.028)
Never married	0.692	0.581***	0.591***	0.659***	0.668***	0.799***	0.806***	0.858**	0.863**
	(0.203)	(0.031)	(0.032)	(0.059)	(0.058)	(0.056)	(0.056)	(0.051)	(0.051)
Lost partner	0.807**	0.607***	0.613***	0.754***	0.768***	0.909**	0.920*	0.947	0.956
	(0.078)	(0.022)	(0.022)	(0.043)	(0.043)	(0.043)	(0.043)	(0.038)	(0.039)
All other situations (base)									
No longer officially together	0.982	1.420***	1.394***	1.305**	1.282**	1.235**	1.225**	1.212**	1.196**
	(0.093)	(0.110)	(0.108)	(0.143)	(0.138)	(0.115)	(0.116)	(0.094)	(0.092)
Newly officially together	1.005	0.656***	0.668***	0.618***	0.627***	0.725***	0.731***	0.731***	0.730***
	(0.093)	(0.049)	(0.050)	(0.056)	(0.056)	(0.072)	(0.072)	(0.056)	(0.056)
Retired (base)									
Employed or self-employed	1.281***	1.552***	1.516***	1.217***	1.185***	1.128**	1.094*	1.123***	1.092**
	(0.061)	(0.053)	(0.051)	(0.060)	(0.058)	(0.055)	(0.054)	(0.042)	(0.041)
Unemployed	0.590***	0.285***	0.294***	0.184***	0.191***	0.211***	0.217***	0.203***	0.210***
	(0.051)	(0.020)	(0.021)	(0.022)	(0.023)	(0.022)	(0.023)	(0.019)	(0.020)
Permanently sick	0.905	0.502***	0.554***	0.431***	0.495***	0.479***	0.549***	0.462***	0.538***
	(0.070)	(0.030)	(0.033)	(0.041)	(0.047)	(0.040)	(0.045)	(0.033)	(0.037)
Homemaker	0.771***	0.623***	0.631***	0.647***	0.653***	0.718***	0.719***	0.724***	0.727***
	(0.051)	(0.027)	(0.027)	(0.044)	(0.044)	(0.041)	(0.041)	(0.035)	(0.035)
Other	0.928	0.704***	0.723***	0.543***	0.558***	0.631***	0.638***	0.602***	0.610***
	(0.079)	(0.050)	(0.051)	(0.068)	(0.069)	(0.064)	(0.065)	(0.056)	(0.056)
Not limited in activities (base)									
Limited	0.741***	0.502***	-	0.614***	-	0.563***	-	0.608***	-
	(0.019)	(0.010)		(0.018)		(0.016)		(0.013)	
Excellent self-perceived health status (base)									
Very good	-	-	0.865***	-	0.956	-	1.055	-	0.942
			(0.043)		(0.083)		(0.099)		(0.064)
Good	-	-	0.589***	-	0.655***	-	0.714***	-	0.669***
			(0.028)		(0.053)		(0.062)		(0.044)
Fair	-	-	0.369***	-	0.446***	-	0.468***	-	0.460***
			(0.018)		(0.037)		(0.041)		(0.031)
Poor	-	-	0.209***	-	0.253***	-	0.287***	-	0.278***
			(0.011)		(0.023)		(0.027)		(0.020)
Austria (base)									
Germany	-	0.755***	0.833**	0.638***	0.704***	0.789***	0.859*	0.782***	0.849***
		(0.061)	(0.067)	(0.054)	(0.058)	(0.063)	(0.068)	(0.044)	(0.047)
Sweden	-	0.890	0.879	1.163	1.136	1.137	1.129	1.146**	1.131*
		(0.076)	(0.075)	(0.109)	(0.105)	(0.107)	(0.105)	(0.074)	(0.072)
Spain	-	0.101***	0.120***	0.108***	0.125***	0.187***	0.213***	0.222***	0.252***
		(0.007)	(0.009)	(0.012)	(0.013)	(0.017)	(0.019)	(0.016)	(0.018)
Italy	-	0.034***	0.040***	0.035***	0.039***	0.095***	0.106***	0.099***	0.110***
		(0.003)	(0.003)	(0.003)	(0.003)	(0.007)	(0.008)	(0.005)	(0.006)
France	-	0.203***	0.229***	0.217***	0.242***	0.342***	0.376***	0.372***	0.406***
		(0.016)	(0.018)	(0.019)	(0.020)	(0.027)	(0.029)	(0.021)	(0.023)
Denmark	-	1.676***	1.631***	2.683***	2.574***	1.602***	1.570***	2.103***	2.050***
		(0.154)	(0.149)	(0.249)	(0.236)	(0.146)	(0.143)	(0.135)	(0.131)
Switzerland	-	1.080	1.058	1.269**	1.228**	1.049	1.027	1.085	1.068
		(0.099)	(0.096)	(0.120)	(0.114)	(0.098)	(0.095)	(0.070)	(0.068)
Belgium	-	0.257***	0.261***	0.401***	0.407***	0.469***	0.468***	0.593***	0.595***
		(0.019)	(0.020)	(0.036)	(0.036)	(0.037)	(0.037)	(0.035)	(0.035)
Czechia	-	0.193***	0.210***	0.155***	0.168***	0.301***	0.315***	0.293***	0.307***
		(0.014)	(0.016)	(0.015)	(0.016)	(0.029)	(0.030)	(0.019)	(0.020)
Poland	-	0.029***	0.036***	0.040***	0.049***	0.095***	0.110***	0.109***	0.125***
		(0.003)	(0.003)	(0.004)	(0.005)	(0.008)	(0.009)	(0.007)	(0.008)
Luxembourg	-	1.012	1.093	0.672***	0.721***	0.902	0.963	0.834**	0.882
		(0.111)	(0.119)	(0.079)	(0.083)	(0.103)	(0.109)	(0.065)	(0.069)
Portugal	-	0.029***	0.038***	0.035***	0.044***	0.095***	0.117***	0.107***	0.130***
		(0.003)	(0.003)	(0.006)	(0.007)	(0.012)	(0.015)	(0.011)	(0.013)
Slovenia	-	0.033***	0.037***	0.052***	0.057***	0.104***	0.111***	0.134***	0.143***
		(0.002)	(0.003)	(0.004)	(0.005)	(0.008)	(0.008)	(0.008)	(0.008)
Estonia	-	0.038***	0.051***	0.046***	0.061***	0.123***	0.157***	0.130***	0.164***
		(0.003)	(0.004)	(0.004)	(0.005)	(0.009)	(0.012)	(0.007)	(0.009)
Croatia	-	0.013***	0.014***	0.021***	0.024***	0.059***	0.063***	0.076***	0.083***
		(0.001)	(0.001)	(0.002)	(0.002)	(0.005)	(0.005)	(0.004)	(0.005)
2015 (base)									
2017	-	-	-	-	-	1.285***	1.285***	1.248***	1.250***
						(0.046)	(0.047)	(0.035)	(0.035)
2020	-	-	-	-	-	1.165***	1.132***	1.006	0.982
						(0.041)	(0.040)	(0.025)	(0.025)
2022	-	-	-	-	-	1.294***	1.264***	1.092***	1.069**
						(0.048)	(0.047)	(0.029)	(0.029)
Number of observations	50,912	164,331	164,331	164,286	164,286	164,286	164,286	164,286	164,286
Number of respondents	15,572	58,731	58,731	58,701	58,701	-	-	-	-
Calibrated longitudinal weights	No	No	No	Yes	Yes	Yes	Yes	Yes	Yes
Panel structure	Yes	Yes	Yes	Yes	Yes	No	No	No	No
Effects	Fixed	Random	Random	Random	Random	-	-	-	-
Dependent categorical variable	Binary	Binary	Binary	Ordered	Ordered	Binary	Binary	Ordered	Ordered
Non-zero panel level variance	-	Yes	Yes	Yes	Yes	-	-	-	-
Errors in parentheses	Standard	Robust	Robust	Robust	Robust	Cluster	Cluster	Cluster	Cluster

All estimators are logistic estimators reporting odds ratios. Financial distress as binary variable rates 0 Rather difficult, and 1 Rather easy to make ends meet. Financial distress as a categorical variable rates 1 With great difficulty, 2 With some difficulty, 3 Fairly easily, and 4 Easily to make ends meet. Cluster refers to cluster standard errors which allow intragroup correlation, i.e. within respondent. The panel structure relies on the respondent’s unique identifier and the survey year. Regression output relies on the first imputation provided by SHARE. Note that for the second column, 43,159 respondents or 113,419 observations are omitted because of all positive or all negative outcomes, i.e. respondents that consistently report the same value for the dependent variable over time.

*** p < 0.01,

** p < 0.05,

* p < 0.10

**Table 3:** Estimation results for Financial distress in relation to the Relationship status

VARIABLES	(1)	(2)	(3)	(4)	(5)	(6)	(7)	(8)	(9)
Age	1.117***	1.056***	1.058***	1.051***	1.054***	1.033***	1.036***	1.030***	1.033***
	(0.004)	(0.002)	(0.002)	(0.003)	(0.003)	(0.002)	(0.002)	(0.002)	(0.002)
Years of education	0.990	1.125***	1.116***	1.128***	1.120***	1.074***	1.069***	1.076***	1.071***
	(0.013)	(0.004)	(0.004)	(0.006)	(0.006)	(0.005)	(0.005)	(0.004)	(0.004)
Male (base)									
Female	-	1.064**	1.045*	1.070	1.060	1.048	1.041	1.043	1.035
		(0.027)	(0.027)	(0.044)	(0.043)	(0.037)	(0.037)	(0.028)	(0.028)
No income (base)									
Below country average income	1.013	0.877***	0.869***	0.906***	0.893***	1.029	1.031	1.029	1.029
	(0.023)	(0.018)	(0.018)	(0.030)	(0.030)	(0.046)	(0.046)	(0.037)	(0.037)
Above country average income	1.360***	2.524***	2.466***	2.542***	2.483***	3.384***	3.351***	3.210***	3.168***
	(0.039)	(0.065)	(0.064)	(0.102)	(0.100)	(0.170)	(0.170)	(0.125)	(0.124)
No liabilities (base)									
Below country average liabilities	0.647***	0.378***	0.382***	0.382***	0.385***	0.366***	0.373***	0.373***	0.380***
	(0.029)	(0.014)	(0.014)	(0.022)	(0.022)	(0.019)	(0.019)	(0.016)	(0.016)
Above country average liabilities	0.610***	0.411***	0.407***	0.389***	0.394***	0.338***	0.343***	0.399***	0.406***
	(0.037)	(0.021)	(0.021)	(0.029)	(0.029)	(0.026)	(0.027)	(0.024)	(0.025)
No mortgage (base)									
Mortgage	0.847***	0.857***	0.844***	0.728***	0.727***	0.761***	0.753***	0.767***	0.761***
	(0.043)	(0.033)	(0.033)	(0.041)	(0.041)	(0.047)	(0.047)	(0.034)	(0.034)
Not single (base)									
Single	0.625***	0.454***	0.458***	0.570***	0.582***	0.727***	0.735***	0.779***	0.785***
	(0.050)	(0.013)	(0.013)	(0.027)	(0.027)	(0.028)	(0.028)	(0.025)	(0.025)
Relationship status unchanged (base)									
Hooked up	0.960	0.655***	0.665***	0.751***	0.769***	0.798**	0.817**	0.808***	0.827***
	(0.073)	(0.040)	(0.041)	(0.071)	(0.071)	(0.079)	(0.079)	(0.059)	(0.060)
Back single	1.011	1.469***	1.441***	1.267**	1.249**	1.112	1.113	1.138	1.143*
	(0.081)	(0.096)	(0.095)	(0.141)	(0.135)	(0.100)	(0.099)	(0.091)	(0.090)
Retired (base)									
Employed or self-employed	1.294***	1.580***	1.544***	1.232***	1.200***	1.138***	1.103**	1.132***	1.100**
	(0.062)	(0.054)	(0.052)	(0.061)	(0.059)	(0.056)	(0.054)	(0.042)	(0.041)
Unemployed	0.594***	0.288***	0.296***	0.186***	0.192***	0.213***	0.219***	0.204***	0.210***
	(0.051)	(0.020)	(0.021)	(0.023)	(0.023)	(0.022)	(0.023)	(0.019)	(0.020)
Permanently sick	0.907	0.504***	0.557***	0.435***	0.499***	0.480***	0.552***	0.463***	0.538***
	(0.071)	(0.030)	(0.033)	(0.042)	(0.047)	(0.040)	(0.045)	(0.033)	(0.037)
Homemaker	0.769***	0.628***	0.636***	0.659***	0.666***	0.736***	0.738***	0.742***	0.744***
	(0.051)	(0.027)	(0.027)	(0.044)	(0.044)	(0.042)	(0.041)	(0.036)	(0.036)
Other	0.930	0.730***	0.749***	0.564***	0.580***	0.668***	0.676***	0.634***	0.643***
	(0.079)	(0.052)	(0.053)	(0.070)	(0.072)	(0.068)	(0.069)	(0.059)	(0.059)
Not limited in activities (base)									
Limited	0.741***	0.502***	-	0.613***	-	0.560***		0.605***	
	(0.019)	(0.010)		(0.018)		(0.016)		(0.013)	
Excellent self-perceived health status (base)									
Very good	-	-	0.868***	-	0.964	-	1.073	-	0.953
			(0.043)		(0.083)		(0.100)		(0.064)
Good	-	-	0.589***	-	0.658***	-	0.721***	-	0.674***
			(0.028)		(0.053)		(0.062)		(0.044)
Fair	-	-	0.369***	-	0.448***	-	0.471***	-	0.462***
			(0.018)		(0.037)		(0.041)		(0.031)
Poor	-	-	0.208***	-	0.253***	-	0.287***	-	0.278***
			(0.011)		(0.023)		(0.027)		(0.020)
Austria (base)									
Germany	-	0.764***	0.843**	0.653***	0.721***	0.804***	0.876*	0.797***	0.865**
		(0.062)	(0.068)	(0.056)	(0.060)	(0.064)	(0.069)	(0.045)	(0.049)
Sweden	-	0.893	0.882	1.165	1.138	1.137	1.129	1.146**	1.131*
		(0.077)	(0.076)	(0.110)	(0.106)	(0.107)	(0.105)	(0.074)	(0.073)
Spain	-	0.105***	0.125***	0.114***	0.132***	0.195***	0.223***	0.230***	0.261***
		(0.008)	(0.009)	(0.012)	(0.014)	(0.017)	(0.020)	(0.017)	(0.019)
Italy	-	0.036***	0.042***	0.037***	0.042***	0.100***	0.112***	0.103***	0.115***
		(0.003)	(0.003)	(0.003)	(0.004)	(0.008)	(0.008)	(0.006)	(0.006)
France	-	0.208***	0.234***	0.222***	0.248***	0.351***	0.386***	0.381***	0.416***
		(0.016)	(0.018)	(0.019)	(0.021)	(0.028)	(0.030)	(0.022)	(0.024)
Denmark	-	1.665***	1.619***	2.697***	2.588***	1.607***	1.576***	2.108***	2.056***
		(0.153)	(0.149)	(0.253)	(0.239)	(0.147)	(0.144)	(0.136)	(0.133)
Switzerland	-	1.053	1.032	1.235**	1.197*	1.025	1.004	1.066	1.050
		(0.097)	(0.094)	(0.118)	(0.112)	(0.096)	(0.093)	(0.069)	(0.068)
Belgium	-	0.259***	0.264***	0.406***	0.412***	0.474***	0.472***	0.599***	0.600***
		(0.020)	(0.020)	(0.037)	(0.037)	(0.038)	(0.037)	(0.036)	(0.036)
Czechia	-	0.194***	0.210***	0.158***	0.171***	0.308***	0.322***	0.298***	0.312***
		(0.014)	(0.016)	(0.016)	(0.017)	(0.030)	(0.031)	(0.020)	(0.021)
Poland	-	0.031***	0.038***	0.044***	0.053***	0.103***	0.119***	0.116***	0.134***
		(0.003)	(0.003)	(0.004)	(0.005)	(0.009)	(0.010)	(0.007)	(0.009)
Luxembourg	-	1.033	1.114	0.691***	0.742**	0.915	0.977	0.850**	0.900
		(0.114)	(0.122)	(0.082)	(0.086)	(0.106)	(0.112)	(0.067)	(0.071)
Portugal	-	0.030***	0.039***	0.036***	0.046***	0.098***	0.122***	0.110***	0.134***
		(0.003)	(0.004)	(0.006)	(0.008)	(0.013)	(0.015)	(0.011)	(0.014)
Slovenia	-	0.035***	0.039***	0.056***	0.061***	0.111***	0.118***	0.141***	0.151***
		(0.003)	(0.003)	(0.005)	(0.005)	(0.008)	(0.009)	(0.008)	(0.009)
Estonia	-	0.038***	0.051***	0.047***	0.061***	0.126***	0.161***	0.133***	0.167***
		(0.003)	(0.004)	(0.004)	(0.005)	(0.009)	(0.012)	(0.007)	(0.009)
Croatia	-	0.013***	0.015***	0.023***	0.026***	0.063***	0.068***	0.081***	0.088***
		(0.001)	(0.001)	(0.002)	(0.002)	(0.005)	(0.005)	(0.005)	(0.005)
2015 (base)									
2017	-	-	-	-	-	1.276***	1.276***	1.240***	1.242***
						(0.046)	(0.046)	(0.035)	(0.035)
2020	-	-	-	-	-	1.156***	1.123***	1.001	0.977
						(0.040)	(0.040)	(0.025)	(0.024)
2022	-	-	-	-	-	1.279***	1.249***	1.080***	1.058**
						(0.047)	(0.046)	(0.029)	(0.028)
Number of observations	50,912	164,331	164,331	164,286	164,286	164,286	164,286	164,286	164,286
Number of respondents	15,572	58,731	58,731	58,701	58,701	-	-	-	-
Calibrated longitudinal weights	No	No	No	Yes	Yes	Yes	Yes	Yes	Yes
Panel structure	Yes	Yes	Yes	Yes	Yes	No	No	No	No
Effects	Fixed	Random	Random	Random	Random	-	-	-	-
Dependent categorical variable	Binary	Binary	Binary	Ordered	Ordered	Binary	Binary	Ordered	Ordered
Non-zero panel level variance	-	Yes	Yes	Yes	Yes	-	-	-	-
Errors in parentheses	Standard	Robust	Robust	Robust	Robust	Cluster	Cluster	Cluster	Cluster

All estimators are logistic estimators reporting odds ratios. Financial distress as binary variable rates 0 Rather difficult, and 1 Rather easy to make ends meet. Financial distress as a categorical variable rates 1 With great difficulty, 2 With some difficulty, 3 Fairly easily, and 4 Easily to make ends meet. Cluster refers to cluster standard errors which allow intragroup correlation, i.e. within respondent. The panel structure relies on the respondent’s unique identifier and the survey year. Regression output relies on the first imputation provided by SHARE. Note that for the second column, 43,159 respondents or 113,419 observations are omitted because of all positive or all negative outcomes, i.e. respondents that consistently report the same value for the dependent variable over time.

*** p < 0.01,

** p < 0.05,

* p < 0.10

## Data Availability

To provide insight into the econometric analysis completed in this article, do- and log-files of Stata are available via https://github.com/hansgevers/financialdistressandmarriage.
